# A58 CHARACTERIZATION OF THE 37/67 KDA LAMININ RECEPTOR IN COLORECTAL CANCER CELLS

**DOI:** 10.1093/jcag/gwab049.057

**Published:** 2022-02-21

**Authors:** G Cloutier, T Khalfaoui, J Beaulieu

**Affiliations:** Universite de Sherbrooke Faculte de Medecine et des Sciences de la Sante, Sherbrooke, QC, Canada

## Abstract

**Background:**

The interaction between cells and extracellular matrix components like laminin or elastin is a crucial step in cancer development and the metastasis process. The 67 kDa Laminin Receptor (67LR) was one of the first cell surface receptors for laminin to be discovered in the 1980s. The 67LR has been reported to be the cytoplasmic 37 kDa small ribosomal subunit protein RPSA (40S ribosomal protein SA) combined with another yet to be identified component to become the 67 kDa membrane receptor. The mechanism by which the ribosomal protein becomes the 67LR membrane receptor is still unclear, but it is presumed that the process involves post-translational modifications combined with homo or hetero-dimerization with non-associated ribosomal proteins. Interestingly, the 67LR confers aggressiveness and a poor prognosis to a wide variety of cancers.

**Aims:**

The aim of this study was to confirm the overexpression of 67LR at the membrane of colorectal cancer cells (CRC) and to identify its homo or hetero-dimerization partners.

**Methods:**

To detect the expression of 67LR in colorectal cancer we performed indirect immunofluorescence on tissues from normal and diseased colons. To confirm the presence of 67LR at the membrane of CRC cells we used a cellular fractionation protocol combined with ultracentrifugation and detergent treatment to separate ribosome-containing fractions from the membranes to isolate membrane associated 67LR-related components. Mass spectrometry analysis to study the molecular identity of 67LR was performed on immunoreactive bands corresponding to RPSA and 67LR from different subcellular fractions.

**Results:**

Immunolocalization of 67LR revealed an overexpression in colorectal cancer tissues. Following analysis by western blotting a 67 kDa immunoreactive protein was found in the supernatant of the 210,000 x g centrifugation while a 37 kDa protein was detected in the pellet solubilized with detergent, suggesting that the 37 kDa form is associated with the membrane. Furthermore, mass spectrometry analysis of the 67 kDa immunoreactive band present in the soluble fraction did not identify any RPSA-related peptides but found a 67 kDa elastin binding receptor (67EBP) related peptide, an elastin receptor that can also bind laminin. The identity of the 67EBP was then confirmed using a shRNA knockdown approach.

**Conclusions:**

These results suggest a possible confusion between the 67 kDa laminin receptor (67LR) and 67 kDa elastin receptor (67EBP) and the possibility that the 37 kDa protein acts as a cell membrane laminin receptor.

**Funding Agencies:**

CIHR

